# Stage-Dependent Reorganization of Inflammatory Biomarker Networks During Acute Exacerbations of COPD

**DOI:** 10.3390/life16071173

**Published:** 2026-07-16

**Authors:** Larisa Alexandra Rus, Romana Olivia Popețiu, Simona Maria Borta, Nicolae Cătălin Hreniuc, Adrian Silviu Crișan, Carla Melania Tamaș, Evelin-Anda Laza, Paula Alexandra Vulciu, Oana Știrbu, Cecilia Avram, Denisa Goldiș, Darius Radu Roman, Alexandru Chioreanu, Radmila-Anca Bugari, Dana Zdremțan, Cristina Georgiana Firu, Imola Donath-Miklos, Maria Pușchiță

**Affiliations:** 1 Department of Internal Medicine, Faculty of Medicine, “Vasile Goldiș” Western University of Arad, Bulevardul Revoluției 94, 310025 Arad, Romania; larisa_gal@yahoo.com (L.A.R.); popetiur@gmail.com (R.O.P.); oanastirbu66@yahoo.com (O.Ș.); mpuschita.mp@gmail.com (M.P.); 2 Arad County Emergency Clinical Hospital, Str. Andrényi Károly Nr. 24, 310037 Arad, Romania; cata_hr@yahoo.com (N.C.H.); adriancrisan74@yahoo.com (A.S.C.); carla_tamas@yahoo.com (C.M.T.); avramcici02@yahoo.com (C.A.); timisdenisa333@yahoo.com (D.G.); 3 Department of Neurology, Faculty of Medicine, “Vasile Goldiș” Western University of Arad, Bulevardul Revoluției 94, 310025 Arad, Romania; 4 Department of Critical Care and Emergency Medicine, “Vasile Goldiș” Western University of Arad, Bulevardul Revoluției 94, 310025 Arad, Romania; 5 The National Institute of Research—Development for Machines and Installations Designed for Agricultureand Food Industry (INMA), Bulevardul Ion Ionescu de la Brad 6, 013813 Bucharest, Romania; eveline_anda@yahoo.com; 6 Department of Biochemistry, Faculty of Medicine, “Vasile Goldiș” Western University of Arad, Bulevardul Revoluției 94, 310025 Arad, Romania; vulciu.paula@uvvg.ro (P.A.V.); zdremtan.dana@uvvg.ro (D.Z.); 7 Department of Biology and Life Science, Faculty of Medicine, “Vasile Goldiș” Western University of Arad, Bulevardul Revoluției 94, 310025 Arad, Romania; 8 Clinical Laboratory of Medical Analyses, Arad County Emergency Clinical Hospital, Str. Andrényi Károly Nr 2–4, 310037 Arad, Romania; 9 Doctoral School of Biomedical Sciences, Faculty of Medicine, and Pharmacy, University of Oradea, Str. Universității Nr 1, 410087 Oradea, Romania; roman.dariusradu@student.uoradea.ro; 10 Department of Otorinolaryngology, Faculty of Medicine, “Vasile Goldiș” Western University of Arad, Bulevardul Revoluției 94, 310025 Arad, Romania; chioreanu.alexandru@uvvg.ro (A.C.); radmilabugariturcin@gmail.com (R.-A.B.); 11 Department of Heamatology, Faculty of Medicine, “Vasile Goldiș” Western University of Arad, Bulevardul Revoluției 94, 310025 Arad, Romania; paracris17@yahoo.com

**Keywords:** acute exacerbation of COPD, GOLD stages, airflow limitation, C-reactive protein, interleukin-6, procalcitonin, inflammatory network remodeling, biomarker interactions, systemic inflammation, disease stratification

## Abstract

Acute exacerbations of chronic obstructive pulmonary disease (AECOPD) are characterized by intensified systemic inflammation, yet the relationship between inflammatory biomarker profiles and underlying GOLD-defined airflow limitation severity remains incompletely understood. This exploratory cross-sectional study investigated inflammatory and hematological biomarkers across the spectrum of stable-state GOLD-defined airflow limitation severity in patients hospitalized for AECOPD. A total of 100 participants were included, comprising non-COPD controls and patients with mild-to-moderate (GOLD 1–2), severe (GOLD 3), and very severe (GOLD 4) COPD (*n* = 25/group). Absolute leukocyte count, C-reactive protein (CRP), erythrocyte sedimentation rate, interleukin-6 (IL-6), procalcitonin, and hemoglobin were analyzed using non-parametric statistics, ordinal logistic regression, and Spearman correlation analysis. Absolute leukocyte counts, CRP, IL-6, and procalcitonin differed significantly across the airflow limitation continuum (*p* < 0.05). Among all variables examined, CRP was the only biomarker independently associated with increasing GOLD-defined airflow limitation severity (OR = 1.15, 95% CI: 1.01–1.31, *p* = 0.034). Correlation analyses revealed stage-dependent alterations in biomarker interaction patterns, with strong CRP-centered associations observed in GOLD 1–2 progressively weakening in advanced disease stages. These findings suggest that COPD severity is associated not only with quantitative changes in inflammatory biomarkers but also with progressive reorganization of inflammatory biomarker relationships, supporting the potential value of CRP as a complementary marker of disease stratification during AECOPD.

## 1. Introduction

### 1.1. COPD as a Systemic Inflammatory Disease

Acute exacerbations of chronic obstructive pulmonary disease (AECOPDs) are clinical events characterized by a sudden worsening of respiratory symptoms beyond the patient’s baseline condition and the need for treatment escalation [1,2,3,4,5]. These episodes contribute substantially to morbidity, mortality, and healthcare expenditure, accounting for a considerable proportion of COPD-related hospitalizations and associated costs [6,7]. Although the severity of airflow limitation is closely linked to disease progression, exacerbation risk, and long-term outcomes [8,9,10], it is not the primary determinant of acute therapeutic decision-making during exacerbations [11,12].

Spirometric assessment remains a cornerstone of COPD severity classification through the Global Initiative for Chronic Obstructive Lung Disease (GOLD) framework, which relies on post-bronchodilator Forced Expiratory Volume in one second (FEV1) expressed as a percentage of the predicted value [12,13,14]. Despite its central role, spirometry is effort-dependent, technically demanding, and may not fully capture the biological heterogeneity of COPD, particularly during acute exacerbations [11].

COPD is increasingly recognized as a systemic inflammatory disorder extending beyond the respiratory tract. Persistent activation of innate and adaptive immune pathways contributes not only to pulmonary damage but also to the development of systemic manifestations and comorbidities [10,15].

### 1.2. Inflammatory and Hematological Biomarkers in AECOPD

During AECOPD, inflammatory responses become amplified, resulting in measurable alterations of circulating inflammatory and hematological biomarkers. Several routinely available biomarkers, including absolute leukocyte count, C-reactive protein (CRP), interleukin-6 (IL-6), procalcitonin (PCT), erythrocyte sedimentation rate (ESR), and hemoglobin, have been investigated in relation to exacerbation severity, infection burden, hospitalization risk, and clinical outcomes [15,16,17,18,19,20,21,22,23,24,25].

Absolute leukocyte count reflects activation of innate immune responses and has been associated with adverse clinical outcomes during exacerbations [15,16,17]. CRP remains one of the most extensively studied inflammatory biomarkers in COPD and has been linked to exacerbation burden, readmission risk, mortality, and disease severity [10,11,22,23,24]. IL-6, a key pro-inflammatory cytokine, has been associated with impaired lung function and increased exacerbation frequency [24,26], whereas PCT is primarily used to identify bacterial infections and guide antibiotic therapy [26]. ESR continues to be routinely used in clinical practice despite receiving relatively limited attention in COPD research [18,19], while hemoglobin concentrations have been associated with exacerbation frequency and long-term prognosis [20,21].

Despite extensive investigation of these biomarkers individually, their relationship with underlying GOLD-defined airflow limitation severity remains incompletely characterized. Existing evidence suggests that some inflammatory markers may increase with disease severity, but stage-specific trajectories and interaction patterns remain insufficiently understood.

### 1.3. From Individual Biomarkers to Inflammatory Networks

COPD progression is increasingly viewed as the consequence of complex interactions between inflammatory, immune, and tissue-remodeling pathways rather than the activity of isolated biological mediators. Contemporary systems biology and network medicine frameworks propose that chronic diseases emerge from dynamic alterations within interconnected molecular and cellular networks, where the relationships between biomarkers may be as informative as their absolute concentrations [27,28].

Under this perspective, systemic inflammation represents an integrated biological process involving coordinated interactions among cytokines, acute-phase proteins, leukocyte populations, and downstream effector mechanisms [29,30,31]. Consequently, disease progression may be reflected not only by quantitative changes in individual biomarkers but also by alterations in the structure and strength of biomarker interaction networks. This concept has gained increasing attention in chronic inflammatory diseases and has contributed to a more nuanced understanding of disease heterogeneity and biological endotypes [1,32,33,34].

Nevertheless, most biomarker studies in AECOPD have focused on the diagnostic or prognostic value of individual inflammatory markers, while considerably less attention has been directed toward understanding how inflammatory biomarker relationships evolve across different stages of GOLD-defined airflow limitation severity. Network-oriented approaches remain relatively uncommon despite their potential to reveal coordinated inflammatory patterns [35].

### 1.4. Study Objectives and Hypothesis

The primary objective of this exploratory study was to compare inflammatory and hematological biomarker profiles across different stages of stable-state GOLD-defined airflow limitation severity in patients hospitalized for AECOPD. The secondary objective was to investigate stage-dependent patterns of interaction among inflammatory biomarkers using correlation-based network analysis.

We hypothesized that GOLD-defined airflow limitation severity is associated not only with quantitative alterations in inflammatory and hematological biomarker levels during AECOPD but also with progressive reorganization of inflammatory biomarker interaction patterns. Therefore, the present exploratory study investigated both biomarker profiles and biomarker interaction networks across different stages of stable-state GOLD-defined airflow limitation severity in a well-matched cohort of Romanian patients hospitalized for AECOPD. By integrating conventional biomarker assessment with correlation-based network analysis, we sought to provide a broader perspective on systemic inflammatory responses across the COPD severity spectrum.

## 2. Materials and Methods

### 2.1. Study Design

This was a single-center, observational, exploratory cross-sectional pilot study designed to investigate both inflammatory and hematological biomarker profiles and biomarker interaction patterns across different stages of stable-state GOLD-defined airflow limitation severity. The study was conducted at the Department of Pneumology of the Arad County Emergency Clinical Hospital (SCJU Arad), Romania, between June 2024 and March 2025. Ethical approval was obtained from the Independent Ethics Committee of SCJU Arad (approval No. 38/15 May 2024) and the Ethics Committee of “Vasile Goldiș” Western University of Arad (approval No. 17/26 March 2024). All participants, or their legal caregivers when applicable, provided written informed consent before study inclusion.

The study was designed around two complementary analytical levels. First, inflammatory and hematological biomarker concentrations were compared across different GOLD-defined airflow limitation stages. Second, correlation-based network analyses were performed to evaluate whether relationships among biomarkers varied according to disease severity.

### 2.2. Study Population

To obtain a relatively homogeneous study population, participants were matched for age, sex, area of residence (urban versus rural), and smoking status. Given the substantial overlap in clinical characteristics between GOLD stage 1 and GOLD stage 2 disease, these categories were combined into a single mild-to-moderate airflow limitation group, consistent with common clinical and research practice [36,37].

A total of 100 participants were included and allocated into four groups of equal size (*n* = 25 per group): (i) healthy non-COPD controls; (ii) mild-to-moderate COPD (GOLD stages 1–2); (iii) severe COPD (GOLD stage 3); and (iv) very severe COPD (GOLD stage 4). The selected sample size was consistent with practical recommendations for exploratory pilot studies [38].

All patients were treated according to the same institutional protocol, with a standard treatment duration of approximately 7 days. Therefore, no meaningful variability was available to report as mean ± SD.

The non-COPD control group consisted of ambulatory patients referred for the evaluation of non-respiratory conditions, most commonly hypertension or gastrointestinal complaints. These individuals did not require hospitalization and had no history or clinical evidence of chronic respiratory disease or acute respiratory symptoms at the time of enrollment. Also, they signed the informed consent form prior the study inclusion.

### 2.3. Inclusion and Exclusion Criteria

Inclusion criteria for COPD patients were: (i) aged 40 years or older; (ii) prior diagnosis of COPD based on spirometric criteria according to GOLD guidelines (post-bronchodilator FEV_1_/FVC < 0.70) and classified as GOLD stage 1, 2, 3, or 4 post-AECOPD; (iii) admission to hospital due to AECOPD—defined as a sudden and significant worsening of a patient’s baseline respiratory symptoms, such as increased dyspnea (shortness of breath), cough, and/or sputum production (often with changes in volume or purulence), that requires a modification/intensification of their usual maintenance therapy [12,39]; (iv) venous blood sampling within 24 h of hospital admission and prior to the administration of systemic corticosteroids or antibiotics; and (v) availability of complete biomarker data (blood absolute leukocyte count, CRP, IL-6, etc.) and key demographic parameters (age, smoking status, origin area).

Patients were excluded from the study if they met any of the following conditions: (i) acute respiratory infection at the time of blood sampling unrelated to COPD exacerbation. Patients with radiographic evidence of pneumonia or positive sputum microbiological findings were excluded. Viral respiratory infections were excluded based on negative rapid antigen tests for SARS-CoV-2 and influenza A/B, whereas bacterial respiratory infections were excluded based on negative sputum culture results together with the absence of radiographic evidence of pneumonia. (ii) Co-existing chronic lung diseases (e.g., pulmonary fibrosis, active tuberculosis, lung cancer). (iii) Concomitant asthma, asthma-COPD overlap (ACO), or fixed airway obstruction due to asthma. (iv) Alternative causes of acute dyspnea, including heart failure and pulmonary embolism. (v) Active extrapulmonary infection (e.g., urinary tract infections, sepsis, soft tissue infections). (vi) Known active autoimmune/rheumatologic conditions (e.g., rheumatoid arthritis, lupus). (vii) Systemic corticosteroids or immunosuppressive therapies within the past 30 days prior to the acute admission. (viii) Severe uncontrolled systemic diseases (e.g., end-stage renal disease, severe liver disease). (ix) Active malignancy within the last five years. (x) Recent major surgery or trauma within the past three months. (xi) Patients younger than 40 years of age were excluded from both the COPD and non-COPD groups. This age threshold was selected to obtain a more homogeneous study population representative of the typical clinical presentation of COPD. (xii) Patients with cord pulmonale, heart failure, or pulmonary embolism to minimize potential confounding effects of these conditions on inflammatory biomarker profiles during AECOPD.

All subjects (or their caregivers) signed an informed consent form prior to study inclusion.

### 2.4. Biomarker Assessment and Spirometric Classification

Venous blood samples were collected from AECOPD patients within 24 h of hospital admission. Absolute leukocyte counts and hemoglobin concentrations were determined on EDTA-anticoagulated whole blood using the SYSMEX XN-1500 analyzer (Sysmex Corporation, Kobe, Japan). Erythrocyte sedimentation rate was determined via the Westergren method. Serum CRP was measured via high-sensitivity immunoturbidimetric assay with a Cobas Pro Biochemistry analyzer (F. Hoffmann-La Roche Ltd., Basel, Switzerland). Serum concentrations of IL-6 and procalcitonin were quantified using electrochemiluminescence immunoassay (COBAS e 601) on the Cobas Pro Bio platform (F. Hoffmann-La Roche Ltd., Basel, Switzerland). All analyses were run within 1–2 h of sample collection in an ISO-accredited laboratory, with all samples analyzed in triplicate.

These variables were selected because they collectively represent complementary components of systemic inflammatory activity, including acute-phase response, cytokine signaling, leukocyte activation, infection-related pathways, and hematological status.

All enrolled COPD patients had undergone spirometry within one year prior to the index AECOPD episode. For study purposes, formal diagnostic spirometry used for GOLD classification was repeated uniformly 2–4 months after recovery from the acute exacerbation, once a stable clinical condition had been achieved. No clinically significant changes in airflow limitation severity were observed between the pre-exacerbation and follow-up spirometry that resulted in a change of GOLD stage.

This clinical timeline aligns with international consensus guidelines regarding the optimal recovery period required to establish accurate baseline pulmonary function measurements after an acute inflammatory event [31,32]. Spirometry results, specifically the post-bronchodilator Forced Expiratory Volume in one second (FEV_1_) expressed as a percentage of the predicted value, were applied to establish the structural severity groups according to the Global Initiative for Chronic Obstructive Lung Disease (GOLD) classification framework. Accordingly, stable-state GOLD classification was used as an indicator of underlying chronic airflow limitation severity rather than acute respiratory status at hospital admission.

### 2.5. Statistical Analysis

#### 2.5.1. Group Comparisons

Inter-group homogeneity was assessed using the Kruskal–Wallis test for age and the Chi-square test for sex, area of residence, and smoking status. Differences in biomarker values across airflow limitation groups were evaluated using the Kruskal–Wallis test. When significant differences were identified, Dunn’s post hoc tests with Bonferroni correction were performed using the non-COPD group as reference. Epsilon-squared (ε^2^) was used as a measure of non-parametric effect size and interpreted as small (0.01–0.05), moderate (0.06–0.13), or large (>0.14).

#### 2.5.2. Association with GOLD-Defined Airflow Limitation Severity

Biomarkers demonstrating significant between-group differences were further evaluated using ordinal logistic regression to determine their association with GOLD-defined airflow limitation severity. Ordinal logistic regression was selected because the outcome variable (GOLD-defined airflow limitation severity) consisted of naturally ordered categories (non-COPD, GOLD 1–2, GOLD 3, and GOLD 4), making this approach appropriate for evaluating the independent association between inflammatory biomarkers and increasing airflow limitation severity. The dependent variable consisted of four ordered categories: 0, non-COPD; 1, mild-to-moderate COPD (GOLD 1–2); 2, severe COPD (GOLD 3); and 3, very severe COPD (GOLD 4).

Multicollinearity was assessed using variance inflation factors (VIF). The proportional odds assumption was evaluated using the Brant test. Given the exploratory nature of the study and the relatively limited sample size, regression findings should be interpreted as hypothesis-generating. The analysis included 100 observations and nine estimated parameters, corresponding to an events-per-variable ratio of 11.1, which is considered the lower acceptable boundary for stable ordinal logistic regression models [40]. Additional covariates were therefore not introduced to avoid model overparameterization.

#### 2.5.3. Correlation-Based Network Analysis

To explore stage-dependent biomarker interaction patterns, Spearman correlation analyses were performed separately within each airflow limitation group. Correlations were calculated between biomarkers identified as significant in the primary analyses and the remaining inflammatory and hematological parameters. Correlation strength was interpreted as moderate (ρ = 0.40–0.59), strong (ρ = 0.60–0.79), and very strong (ρ ≥ 0.80).

Spearman correlation analysis was selected because the analyzed biomarker distributions were non-parametric and the primary objective was to explore pairwise relationships among inflammatory biomarkers within each airflow limitation group. The resulting correlation matrices were used as exploratory representations of biomarker interaction patterns, allowing qualitative comparison of the overall correlation structure across different levels of GOLD-defined airflow limitation severity.

Because the study aimed to investigate not only individual biomarker alterations but also their relationships across the COPD severity spectrum, the resulting correlation matrices were further interpreted as correlation-based representations of inflammatory biomarker interaction networks.

A two-tailed *p*-value < 0.05 was considered statistically significant for all analyses.

### 2.6. Artificial Intelligence Disclosure

Generative artificial intelligence (GenAI) tools, including ChatGPT (OpenAI GPT-5.5, San Francisco, CA, USA), were used exclusively to assist with language editing, manuscript structuring, conceptual organization, and figure planning during manuscript preparation. No artificial intelligence system was used for data collection, data extraction, data curation, statistical analysis, interpretation of results, generation of scientific conclusions, or manuscript peer-review activities. All scientific content, methodological decisions, statistical analyses, visual representations, and final interpretations were independently reviewed, verified, and approved by the authors.

The authors assume full responsibility for the accuracy, integrity, originality, and scientific validity of all material presented in this manuscript.

## 3. Results

### 3.1. Study Population Characteristics

Sociodemographic characteristics of the study population are presented in Table 1. Distributions of patients in terms of sex, area of residence, and smoking status were similar across the airflow limitation continuum (Chi-square test, *p* ≥ 0.500).

A total of 75 patients with AECOPD and 25 non-COPD controls were included in the study. Each airflow limitation category contained 25 participants, ensuring balanced group sizes throughout the analysis. Among patients hospitalized for AECOPD, 18% required non-invasive ventilation during admission, whereas only 6% required intensive care unit management. No patients underwent invasive mechanical ventilation. Patients admitted to the intensive care unit received the same pharmacological treatment as those managed in the general ward. The only difference was the administration of non-invasive ventilation according to clinical indications. These findings indicate that the cohort predominantly represented moderate-to-severe exacerbation episodes managed without advanced ventilatory support.

### 3.2. Biomarker Profiles Across GOLD-Defined Airflow Limitation Stages

Inflammatory and hematological biomarker distributions across the airflow limitation continuum are presented in Table 2. Overall, COPD patients exhibited higher inflammatory biomarker levels than non-COPD controls. Absolute leukocyte count and CRP demonstrated a progressive increase with advancing GOLD-defined airflow limitation severity, whereas IL-6 displayed a non-linear pattern characterized by peak concentrations in severe COPD (GOLD 3). Procalcitonin levels were elevated in COPD patients compared with controls but remained relatively stable across advanced disease stages. In contrast, hemoglobin concentrations showed minimal variation throughout the airflow limitation spectrum.

Age distribution was comparable across study groups. Significant between-group differences were observed for absolute leukocyte count, CRP, IL-6, and procalcitonin, with effect sizes ranging from moderate to large. Post hoc analyses demonstrated significantly higher absolute leukocyte counts, CRP levels, and procalcitonin concentrations in COPD patients compared with non-COPD controls. Elevated IL-6 concentrations were observed predominantly in patients with severe airflow limitation (GOLD 3). No statistically significant differences were identified for ESR or hemoglobin.

### 3.3. Association Between Biomarkers and Airflow Limitation Severity

The proportional odds assumption was satisfied for the ordinal logistic regression model (Brant test, *p* = 0.579), and no individual predictor violated this assumption (all *p* ≥ 0.210). Variance inflation factor values remained below 5 for all variables, indicating the absence of significant multicollinearity. The results of the ordinal logistic regression analysis are presented in Table 3.

Among the biomarkers evaluated, serum CRP was the only variable independently associated with increasing stable-state GOLD-defined airflow limitation severity (OR = 1.15, 95% CI: 1.01–1.31, *p* = 0.034). Although the magnitude of the association was modest, this finding suggests that CRP may reflect the degree of airflow limitation observed after recovery from the acute exacerbation, rather than serving as a direct surrogate of long-term disease progression. Accordingly, CRP should be interpreted as a complementary inflammatory marker associated with spirometric severity in the context of AECOPD rather than as a standalone marker of COPD progression.

In contrast, absolute leukocyte count, ESR, IL-6, procalcitonin, and hemoglobin did not demonstrate independent associations with GOLD-defined airflow limitation severity after multivariable adjustment. Given the exploratory nature of the study, these findings should be interpreted as hypothesis-generating and warrant validation in larger cohorts.

Because individual biomarker concentrations provide only a partial representation of systemic inflammatory activity, we next examined whether relationships among biomarkers differed across stages of GOLD-defined airflow limitation severity.

### 3.4. Stage-Dependent Reorganization of Inflammatory Biomarker Networks

Correlation-based biomarker interaction patterns differed substantially across the airflow limitation continuum (Table 4). In non-COPD controls, CRP was moderately associated with absolute leukocyte count and IL-6 and strongly associated with ESR, indicating a relatively coherent inflammatory profile.

The most interconnected biomarker network was observed in patients with mild-to-moderate COPD (GOLD 1–2). In this group, CRP demonstrated very strong positive associations with ESR, IL-6, and procalcitonin, alongside a moderate inverse relationship with hemoglobin. These findings suggest a highly coordinated inflammatory response during the earlier stages of airflow limitation.

In severe COPD (GOLD 3), the overall network structure became less interconnected. While significant associations between CRP and absolute leukocyte count, ESR, and procalcitonin remained present, the previously observed strong relationship between CRP and IL-6 was no longer detected. This pattern suggests a partial reorganization of inflammatory biomarker interactions with increasing disease severity.

**Figure 1:**
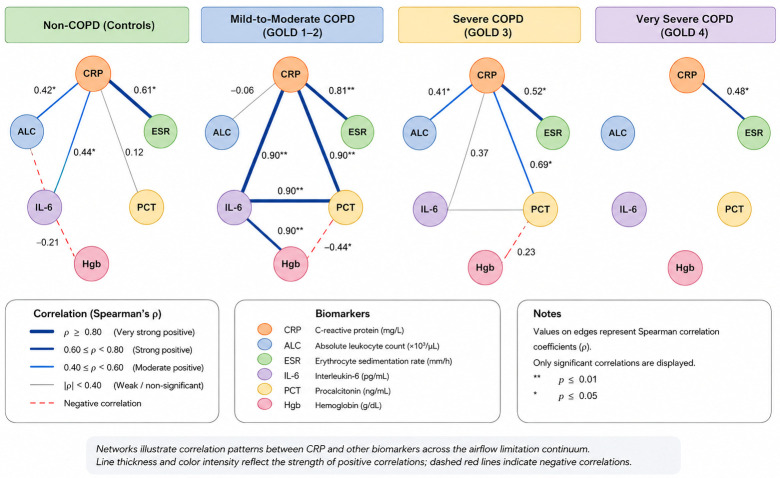
Correlation-based evidence of progressive inflammatory network remodeling across COPD severity stages. Correlation-based representations of CRP-centered inflammatory biomarker interaction patterns across the airflow limitation continuum. Nodes represent inflammatory and hematological biomarkers, whereas edges indicate statistically significant Spearman correlations between CRP and the remaining variables within each study group. Edge thickness is proportional to correlation strength. Mild-to-moderate COPD (GOLD 1–2) exhibited the most interconnected profile, characterized by strong positive associations between CRP, ESR, IL-6, and procalcitonin. Network complexity progressively decreased with increasing GOLD-defined airflow limitation severity, with fewer significant CRP-centered associations observed in severe COPD (GOLD 3) and only ESR remaining significantly associated with CRP in very severe COPD (GOLD 4). These findings suggest stage-dependent remodeling of inflammatory biomarker interaction patterns across the COPD severity spectrum.

The most simplified interaction profile was observed in very severe COPD (GOLD 4). In this group, ESR was the only biomarker that maintained a significant association with CRP, whereas all other previously observed relationships disappeared. Collectively, these findings indicate a progressive reduction in biomarker interconnectedness across the airflow limitation continuum, consistent with stage-dependent remodeling of inflammatory biomarker interaction networks (Figure 1).

## 4. Discussion

### 4.1. Principal Findings

The principal finding of this exploratory cross-sectional study is the stage-dependent reorganization of inflammatory biomarker interaction patterns across GOLD-defined airflow limitation severity in patients hospitalized for AECOPD. Although several inflammatory biomarkers differed significantly across the airflow limitation continuum, serum CRP was the only variable independently associated with increasing stable-state GOLD-defined airflow limitation severity after multivariable adjustment. Beyond this single-marker association, correlation-based analyses revealed marked differences in biomarker interaction patterns across disease stages, ranging from a highly interconnected CRP-centered inflammatory profile in mild-to-moderate COPD to a substantially simplified interaction pattern in very severe disease.

These findings suggest that systemic inflammatory responses during AECOPD may differ not only in magnitude but also in their correlation patterns according to underlying COPD severity. Therefore, the present study supports a broader interpretation of biomarker data in AECOPD, in which individual biomarker concentrations and their interaction patterns are considered complementary dimensions of systemic inflammatory activity.

### 4.2. Stage-Dependent Remodeling of Inflammatory Biomarker Networks

The most interconnected biomarker pattern was observed in patients with mild-to-moderate COPD (GOLD 1–2), where CRP showed very strong positive associations with ESR, IL-6, and procalcitonin, as well as a moderate inverse association with hemoglobin. This pattern is consistent with a coordinated acute-phase inflammatory response involving cytokine signaling, hepatic acute-phase protein synthesis, infection-related pathways, and hematological adaptation. The close relationship between CRP and IL-6 is biologically plausible, as IL-6 is a major regulator of hepatic CRP gene expression through STAT3-mediated signaling [31].

As GOLD-defined airflow limitation severity increased, this coordinated inflammatory profile became progressively less interconnected. In severe COPD (GOLD 3), CRP remained significantly associated with absolute leukocyte count, ESR, and procalcitonin, but the strong CRP–IL-6 relationship observed in mild-to-moderate COPD was no longer detected. In very severe COPD (GOLD 4), ESR was the only biomarker that maintained a significant association with CRP. This progressive reduction in biomarker interconnectedness may reflect a less coordinated pattern of statistical associations among systemic inflammatory biomarkers during AECOPD in patients with more advanced COPD.

From a systems biology and network medicine perspective, chronic diseases are increasingly understood as dynamic disturbances within interconnected molecular and cellular systems rather than as linear alterations of isolated mediators [36,37,41]. In COPD, this concept is particularly relevant because disease progression reflects interactions among immune activation, airway remodeling, parenchymal destruction, systemic inflammation, comorbidities, and repeated exacerbation-related injury [1,40,42]. Previous work has also shown that combining inflammatory biomarkers may reveal clusters or patterns that are not apparent when biomarkers are evaluated individually. In this context, our findings are consistent with the hypothesis that relationships among biomarkers may provide complementary information beyond their absolute concentrations [35].

Several mechanisms may contribute to the observed stage-dependent reorganization of biomarker interactions. Repeated exacerbations, chronic inflammatory stimulation, altered cytokine responsiveness, bacterial or viral triggers, hypoxemia, and systemic comorbidity burden may all modify the relationship between circulating inflammatory mediators. In advanced COPD, greater biological heterogeneity, compartmentalization of inflammation, immune remodeling, and immunosenescence may weaken the statistical coupling between acute-phase proteins, cytokines, leukocyte responses, and infection-related markers [43,44].

It should also be acknowledged that the observed correlation patterns may partly reflect etiological heterogeneity among AECOPD episodes rather than differences attributable solely to GOLD-defined airflow limitation severity. Differences in infectious triggers, inflammatory phenotypes, and other patient-specific factors may have contributed to the observed variability in biomarker interactions. Consequently, because the present study was exploratory and cross-sectional, these interpretations remain hypothesis-generating and should not be considered mechanistic proof.

### 4.3. CRP as a Complementary Marker of GOLD-Defined Airflow Limitation Severity

Serum CRP showed an overall upward trend across the airflow limitation continuum and remained the only biomarker independently associated with stable-state GOLD-defined airflow limitation severity after multivariable adjustment. This association should be interpreted as reflecting spirometric severity following recovery from AECOPD rather than long-term disease progression. Direct comparison with previous studies is challenging because most available reports have focused on CRP in relation to exacerbation severity, infectious phenotypes, readmission, mortality, or FEV1 rather than stage-specific biomarker profiles during AECOPD [7,10,19,21,23,45,46,47,48]. Nevertheless, our findings are broadly consistent with studies reporting increased CRP levels during acute exacerbations and higher systemic inflammatory burden in more advanced COPD [7,45,46,47,48,49].

The biological plausibility of CRP as an integrated marker of systemic inflammation is supported by its position within the acute-phase response. CRP synthesis is largely driven by IL-6-mediated hepatic signaling, but CRP levels may also integrate signals from multiple upstream inflammatory pathways [29,30,31]. This may partly explain why CRP remained independently associated with spirometric airflow limitation severity following recovery from AECOPD, whereas individual upstream or parallel markers such as IL-6, absolute leukocyte count, ESR, procalcitonin, and hemoglobin did not remain independently significant in the regression model.

However, the magnitude of the association was modest and should be interpreted with caution. CRP measured during AECOPD reflects both the underlying chronic inflammatory burden and the intensity of the acute inflammatory episode, including infectious and non-infectious exacerbation components [32,50]. Therefore, CRP should not be interpreted as a surrogate marker of COPD progression or as a substitute for spirometric classification. Rather, it may function as a complementary inflammatory marker associated with spirometric severity in the context of AECOPD, providing additional biological context when assessing hospitalized patients.

### 4.4. Interpretation of Individual Biomarker Profiles

Although serum IL-6 was higher in COPD patients than in controls, its trajectory across GOLD-defined airflow limitation severity was non-linear, with peak concentrations in severe COPD and lower values in very severe disease. Previous studies have reported elevated IL-6 levels in COPD and associations with impaired lung function, exacerbation risk, and adverse outcomes [24,26,33,51,52,53]. However, other reports found no consistent association between IL-6 and GOLD-defined airflow limitation severity despite higher IL-6 concentrations in COPD patients compared with controls [54,55]. Taken together, these findings suggest that IL-6 may reflect systemic inflammatory burden, hypoxemia, symptom severity, and exacerbation phenotype more strongly than spirometric airflow limitation alone.

Absolute leukocyte counts increased with advancing GOLD-defined airflow limitation severity. This may reflect activation of innate immune responses, bone marrow stimulation, bacterial triggers, or repeated tissue injury associated with recurrent exacerbations [56,57,58]. Nevertheless, absolute leukocyte count did not remain independently associated with GOLD-defined severity after multivariable adjustment, suggesting that it may provide overlapping information with other inflammatory variables or primarily reflect the acute exacerbation state.

Procalcitonin was elevated in COPD patients compared with controls but showed a plateau in more advanced stages. This pattern supports its established role as a marker of bacterial or endotoxin-related inflammatory activity during acute respiratory illness rather than as a direct indicator of chronic structural airflow limitation [25,59]. Its strong association with CRP in mild-to-moderate COPD and persistence of association in GOLD 3 may indicate infection-related contribution to the acute inflammatory network during exacerbation.

Hemoglobin concentrations remained relatively stable across the airflow limitation continuum. From a pathophysiological perspective, this may reflect the competing influence of hypoxia-driven erythropoiesis and inflammation-mediated suppression of erythroid activity. Chronic systemic inflammation can promote iron restriction and anemia of inflammation, whereas progressive airflow limitation and hypoxemia may stimulate erythropoietin-mediated red cell production [60]. These opposing mechanisms may partly explain the absence of a clear linear hemoglobin trajectory in the present cohort.

### 4.5. Clinical and Translational Implications

The present findings support the concept that acute inflammatory biomarker assessment may provide information complementary to conventional spirometric classification. Spirometry remains indispensable for diagnosing COPD and defining GOLD-defined airflow limitation severity, but it does not fully capture the biological heterogeneity of COPD, particularly during acute exacerbations. In this context, systemic biomarkers may help characterize the inflammatory state accompanying AECOPD.

More importantly, the observed stage-dependent remodeling of biomarker interaction patterns suggests that correlation-based biomarker networks may provide an additional exploratory framework for studying COPD heterogeneity. Such approaches may help distinguish between patients with preserved coordination of acute inflammatory responses and those exhibiting less coordinated correlation patterns among inflammatory biomarkers. Although this finding has no immediate therapeutic implication, it may contribute to future research on inflammatory endotypes, treatable traits, and biomarker-guided stratification in COPD [61].

### 4.6. Limitations

Several methodological limitations must be acknowledged. First, as a pilot study with a relatively small sample size, the present findings should be interpreted cautiously and require validation in larger prospective cohorts. The cross-sectional observational design precludes causal inference and does not allow characterization of longitudinal biomarker dynamics across disease progression or following treatment. Whether the associations identified here persist over time, predict clinical outcomes such as readmission or mortality, or change after therapeutic intervention remains to be established in prospective longitudinal studies.

Second, exacerbation episodes were characterized clinically, without systematic microbiological workup to distinguish bacterial, viral, and non-infectious etiologies. Because CRP and procalcitonin levels may differ substantially across exacerbation subtypes, etiological heterogeneity may have influenced biomarker values and correlation patterns. Future studies should include microbiological and virological assessment to clarify the specificity of biomarker–GOLD relationships.

Third, prior exacerbation frequency was not systematically collected. This is relevant because the frequently exacerbating phenotype may be associated with persistent systemic inflammation independently of spirometric GOLD-defined airflow limitation severity. Fourth, comorbidity burden was not quantified using a validated index. Although strict exclusion criteria were applied to reduce major confounding, residual confounding cannot be excluded. Additional covariates were not incorporated into the regression model because of the limited sample size and the risk of overparameterization.

Fifth, the correlation-based network analysis should be interpreted cautiously. The study did not include formal graph-theoretical metrics such as centrality, modularity, or clustering coefficients. Therefore, the network interpretation is based on stratified Spearman correlation patterns and should be considered exploratory.

One possible explanation for the progressively weaker biomarker correlations observed across increasing GOLD-defined airflow limitation severity is the growing biological heterogeneity associated with advanced COPD. While earlier disease stages may be characterized by a more coordinated systemic inflammatory response, advanced COPD is influenced by multiple concurrent processes, including chronic inflammation, oxidative stress, immune dysregulation, comorbidities, medication effects, and repeated exacerbations. Consequently, inflammatory biomarkers may become progressively less synchronized, resulting in a less interconnected correlation structure.

Nevertheless, these observations originate from correlation-based analyses and should therefore be interpreted as exploratory rather than mechanistic. The identified biomarker interaction patterns describe statistical associations and do not establish causal biological relationships. Confirmation of these findings will require larger prospective studies incorporating repeated biological sampling before, during, and after AECOPD.

Sixth, participants were recruited from a single tertiary emergency care facility in western Romania, which may limit generalizability to other populations, healthcare systems, environmental exposures, and smoking-related COPD phenotypes.

Another limitation of the present study is that blood eosinophil count and the neutrophil-to-lymphocyte ratio were not included in the predefined biomarker panel. Although both biomarkers have been associated with COPD exacerbations and clinical outcomes, their potential contribution to inflammatory network organization could not be evaluated and warrants investigation in future studies.

In addition, cumulative smoking exposure (pack-years) was not systematically recorded. Although the study groups were matched according to smoking status, differences in smoking intensity may have influenced the inflammatory biomarker profiles and therefore cannot be excluded.

Finally, inflammatory and hematological biomarkers were not reassessed at the time of follow-up spirometry. Because post-exacerbation evaluations were performed exclusively in the outpatient setting, repeated biological sample collection was not feasible. Consequently, the present findings represent a cross-sectional characterization of inflammatory biomarker interactions during AECOPD rather than their longitudinal evolution after clinical recovery.

### 4.7. Future Directions

Future studies should validate these findings in larger, multicenter cohorts with longitudinal biomarker sampling during exacerbation, recovery, and stable disease. Integrating inflammatory biomarkers with microbiological data, exacerbation history, comorbidity profiles, imaging, lung function, and clinical outcomes would allow a more detailed characterization of COPD inflammatory endotypes. In addition, formal network-based approaches using larger biomarker panels, cytokine profiling, omics data, and graph-theoretical metrics may help determine whether inflammatory network remodeling has prognostic or therapeutic relevance in AECOPD.

## 5. Conclusions

This exploratory study demonstrates that systemic inflammatory responses during AECOPD differ not only in magnitude but also in their correlation patterns across GOLD-defined airflow limitation stages. The most notable finding was the progressive reorganization of inflammatory biomarker interaction patterns, characterized by a highly interconnected inflammatory profile in mild-to-moderate COPD and a substantially simplified network architecture in very severe disease.

Among the biomarkers evaluated, serum CRP was the only variable independently associated with increasing stable-state GOLD-defined airflow limitation severity, supporting its potential role as a complementary marker of disease stratification during acute exacerbations. However, the observed network-level changes suggest that individual biomarker concentrations alone may not fully capture the complexity of systemic inflammatory responses in COPD.

Taken together, these findings suggest that systems biology approaches may facilitate the interpretation of inflammatory heterogeneity in COPD and that correlation-based biomarker networks may provide additional insight into disease-associated inflammatory remodeling. Future longitudinal and multicenter studies incorporating larger biomarker panels and formal network-based approaches are needed to determine whether inflammatory network reorganization has prognostic, mechanistic, or therapeutic relevance in COPD.

## Figures and Tables

**Table 1:** Sociodemographic characteristics across airflow limitation continuum.

	Sex		Area of Residence		Smoking Status	
	Female	Male	Urban	Rural	Non-Smoker	Smoker
Non-COPD	10 (40%)	15 (60%)	15 (60%)	10 (40%)	11 (44%)	14 (56%)
Mild-to-moderate COPD	11 (44%)	14 (56%)	11 (44%)	14 (56%)	13 (52%)	12 (48%)
Severe COPD	12 (48%)	13 (52%)	12 (48%)	13 (52%)	14 (56%)	11 (44%)
Very severe COPD	9 (36%)	16 (64%)	9 (36%)	16 (64%)	9 (36%)	16 (64%)

Data are reported as frequencies and percentages (in parentheses). Spirometry for GOLD classification: performed 2–4 months after the index AECOPD episode in all COPD patients. Former smokers were included in the non-smoker category.

**Table 2:** Inflammatory and hematological biomarkers across COPD severity groups.

	Reference Range	N-COPD	MM-COPD	S-COPD	VS-COPD	*p*	ε*^2^*
Age		65	61	67	67	0.182	
		(60; 70)	(57; 72)	(56; 73)	(57; 72)		
ALC (×10^3^/µL)	4.00–10.00	7.98	9.50 *	9.48 *	11.60 *	0.038 *	0.09
		(6.83; 9.19)	(8.02;11.05)	(8.06; 11.05)	(6.89; 13.29)		
CRP (mg/L)	<5.00	4.83	11.91 *	10.72 *	14.40 *	0.020 *	0.11
		(2.65; 7.91)	(2.75; 31.22)	(4.85; 37.84)	(3.68; 54.11)		
ESR (mm/h)	0–30	16	25	15	10	0.425	0.03
		(9; 24)	(10; 30)	(10; 30)	(5; 40)		
IL-6 (pg/mL)	0.00–7.00	5.83	6.90	10.83 *	2.66	0.031 *	0.10
		(3.72; 8.96)	(3.25; 27.17)	(4.34; 21.97)	(2.10; 8.33)		
PCT (ng/mL)	0.00–0.50	0.04	0.06 *	0.07 **	0.06 *	0.009 **	0.14
		(0.03; 0.07)	(0.04; 0.12)	(0.04; 0.16)	(0.04; 0.12)		
Hgb (g/dL)	11.70–17.30	13. 00	14.50	13.80	13.70	0.078	0.06
		(12.70; 14.50)	(12.90; 15.60)	(12.80; 15)	(12.10; 14.30)		

N-COPD, non-COPD; MM, mild-to-moderate COPD (GOLD 1–2); S-COPD, severe COPD (GOLD 3); VS-COPD, very severe COPD (GOLD 4); ε^2^, epsilon-squared (effect size); ALC, absolute leukocyte count; CRP, C-reactive protein; ESR, erythrocyte sedimentation rate; IL-6, interleukin-6; PCT, procalcitonin; Hgb, hemoglobin. The values in the second column correspond to the reference range as per the Romanian standards. Data in the third, fourth, fifth, and sixth columns are given as median values with lower and upper quartiles (in parentheses). Marked values (*) in the seventh column show significant differences across the progressive airflow limitation continuum (Kruskal–Wallis test, **—*p* ≤ 0.01, and *—*p* ≤ 0.05). Marked values (*) in COPD severity groups compared with the non-COPD group (Dunn’s test with Bonferroni correction, **—*p* ≤ 0.01, and *—*p* ≤ 0.05).

**Table 3:** Ordinal logistic regression analysis for variables analyzed.

Predictor	β	SE	OR (95% CI)	z	*p*	VIF
ALC	−0.031	0.190	0.97 (0.67–1.41)	−0.16	0.872	1.87
CRP	0.140	0.066	1.15 (1.01–1.31)	4.49	0.034 *	2.15
ESR	−0.010	0.335	0.99 (0.51–1.91)	−0.03	0.976	1.65
IL-6	−0.031	0.032	0.97 (0.91–1.03)	−0.94	0.345	1.12
PCT	0.131	0.591	1.14 (0.36–3.63)	0.22	0.823	1.30
Hgb	−0.020	0.018	0.98 (0.95–1.02)	−1.12	0.265	1.24

ALC, absolute leukocyte count; CRP, C-reactive protein; ESR, erythrocyte sedimentation rate; IL-6, interleukin-6; PCT, procalcitonin; Hgb, hemoglobin; β, coefficient beta; SE, standard error; OR (95% CI), adjusted odds ratio with 95% confidence interval; Wald (Z), Z-value from the Wald test; *p*, *p* value; VIF, variance inflation factor. Marked values (*) in the last column indicate biomarkers significantly associated with progression of GOLD-defined airflow limitation severity (Ordinal logistic regression, *—*p* ≤ 0.05).

**Table 4:** Correlations between CRP and analyzed biomarkers across COPD severity groups.

	ALC	ESR	IL-6	PCT	Hgb
Non-COPD	0.42 *	0.61 *	0.44 *	0.12	−0.21
Mild-to-moderate COPD (GOLD 1–2)	−0.06	0.81 **	0.90 **	0.90 **	−0.44 *
Severe COPD (GOLD 3)	0.41 *	0.52 *	0.37	0.69 *	0.23
Very severe COPD (GOLD 4)	0.13	0.48 *	−0.08	−0.05	−0.16

ALC, absolute leukocyte count; CRP, C-reactive protein; ESR, erythrocyte sedimentation rate; IL-6, interleukin-6; PCT, procalcitonin; Hgb, hemoglobin. Marked values (*) indicate significant associations (Spearman correlations, **—*p* ≤ 0.01, and *—*p* ≤ 0.05).

## Data Availability

All the data generated or analyzed during this study are included in this published article.

## Abbreviations

The following abbreviations are used in this manuscript:

Abbreviation	Definition
AECOPD	Acute Exacerbation of Chronic Obstructive Pulmonary Disease
ALC	Absolute Leukocyte Count
ACO	Asthma–COPD Overlap
COPD	Chronic Obstructive Pulmonary Disease
CRP	C-Reactive Protein
ESR	Erythrocyte Sedimentation Rate
FEV1	Forced Expiratory Volume in One Second
FVC	Forced Vital Capacity
GenAI	Generative Artificial Intelligence
GOLD	Global Initiative for Chronic Obstructive Lung Disease
Hgb	Hemoglobin
ICU	Intensive Care Unit
IL-6	Interleukin-6
NIV	Non-Invasive Ventilation
OR	Odds Ratio
PCT	Procalcitonin
SCJU Arad	Arad County Emergency Clinical Hospital (Spitalul Clinic Județean de Urgență Arad)
SE	Standard Error
STAT3	Signal Transducer and Activator of Transcription 3
VIF	Variance Inflation Factor
ε^2^	Epsilon-Squared Effect Size
